# Interaction between HLA-B leader peptide variants and cytomegalovirus serostatus is associated with early T cell-mediated rejection in kidney transplantation

**DOI:** 10.3389/fimmu.2026.1713932

**Published:** 2026-02-09

**Authors:** Emma T. M. Peereboom, Jip Jonker, Kirsten Geneugelijk, Arjan D. van Zuilen, Laura B. Bungener, Frans M. Verduyn Lunel, Jan Stephan F. Sanders, Stephan J. L. Bakker, Eric Spierings

**Affiliations:** 1 Center for Translational Immunology, University Medical Center Utrecht, Utrecht University, Utrecht, Netherlands; 2 Division of Nephrology, Department of Internal Medicine, University Medical Center Groningen, University of Groningen, Groningen, Netherlands; 3 Central Diagnostics Laboratory, University Medical Center Utrecht, Utrecht University, Utrecht, Netherlands; 4 Department of Nephrology, University Medical Center Utrecht, Utrecht University, Utrecht, Netherlands; 5 Department of Laboratory Medicine, University Medical Center Groningen, Groningen, Netherlands; 6 Department of Medical Microbiology, University Medical Center Utrecht, Utrecht, Netherlands

**Keywords:** cytomegalovirus (CMV), HLA, kidney transplantation, leader peptides, T-cell-mediated rejection (TCMR)

## Abstract

Mismatches between mature recipient and donor HLA proteins can trigger alloreactivity upon transplantation. Recent studies suggest that also the leader peptide of HLA class I alleles may affect the transplantation outcome. In this retrospective study, we examined the association between the HLA-B leader -21 methionine (M)/threonine (T) dimorphism and T cell-mediated rejection (TCMR) early after kidney transplantation. In a hypothesis-generating cohort of 351 transplants, -21MM recipients experienced significantly increased odds of early TCMR within the first 90 days post-transplantation compared to -21TT recipients (odds ratio (OR) 4.57, 95% confidence interval (CI) 1.87-10.95, p<0.001), irrespective of the donor’s HLA-B leader peptide. This association was most prominent among CMV-seropositive recipients (OR 10.91, 95% CI 3.24-39.24, p<0.001). In an independent cohort (n=936), -21MM CMV-seropositive recipients seemed to be at increased odds of early TCMR. In parallel, among CMV-seropositive recipients, -21MT recipients had a significantly increased likelihood of developing early TCMR (OR 2.74, 95% 1.08-7.88, p=0.04). Combined, CMV-seropositivity in the presence of a -21M leader peptide associated with early TCMR with an OR of 2.95 (95% CI 1.49-5.86, p=0.002). In both cohorts, the effect of the -21M leader peptide was most prominent among recipients mismatched for an HLA-A/-C leader peptide. Conclusively, this study suggests that recipients with an HLA-B -21M leader peptide have increased odds of early TCMR, which is further influenced by the recipient’s CMV serostatus. While the underlying mechanism remains speculative, these findings indicate that the HLA-B leader peptide of the recipient may affect immune regulation and early TCMR after kidney transplantation.

## 1 Introduction

Kidney transplantation is a critical treatment for patients with end-stage renal disease, offering the potential for prolonged survival and improved quality of life (1, 2). However, the long-term success of kidney transplantation is frequently compromised by immune-mediated rejection, including T cell-mediated rejection (TCMR) (3). A key factor in these immune responses is the compatibility of HLA molecules between the donor and recipient. Historically, research on HLA mismatching in transplantation has been focused on the mature proteins encoded by HLA genes, trying to elucidate the risk for rejection (4–7).

Recent studies suggest that other regions of the HLA gene, both coding and non-coding, also play a significant role in transplantation outcomes (8–13). These regions include the non-coding 3’ untranslated region, where single nucleotide polymorphisms may affect the HLA expression levels, thereby increasing the risk of graft-versus-host disease (GvHD) in stem cell transplant recipients (11). HLA leader peptides may influence the outcome of a transplantation as well, despite its absence on the mature HLA protein (12, 13). The HLA leader peptide is a short amino acid sequence, encoded by exon 1, which guides HLA pre-proteins to the endoplasmic reticulum (ER). In the ER, the leader peptide is removed from the pre-protein. While the mature HLA protein is further assembled and directed to the cell surface, leader peptides of HLA class I are able to bind to HLA-E. The expression of HLA-E/leader peptide complexes enhances protection against natural killer (NK) cell-mediated lysis by binding to the inhibitory NKG2A/CD94 receptor complex (14). As leader peptides differ between different HLA class I alleles, leader peptide mismatches between donor and recipient may be present, which may lead to alloreactivity. Indeed, we recently reported a role for HLA-A and -C leader peptide mismatches in the development of TCMR early after transplantation. This effect was found among cytomegalovirus (CMV)-seropositive, but not CMV-seronegative kidney transplant recipients (13).

In the present study, we addressed a potential interaction of CMV serostatus with the HLA-B leader peptide for risk of TCMR. The HLA-B leader peptide displays a dimorphism at position -21, containing either a methionine (M) or threonine (T) at that position. In hematopoietic stem cell transplantation (HSCT), HLA-B leader peptide variants at position -21 may be associated with increased risks of GvHD and other complications (12, 15–21). Yet, findings have been inconsistent, with some studies reporting an effect for HLA-B leader peptide mismatching (12, 16, 17, 19–21), whereas others do not (22–24). In addition, studies about the HLA-B leader peptide dimorphism in solid organ transplantation are lacking. In this study, we aimed to investigate the potential impact of the -21 HLA-B leader peptide dimorphism on the likelihood of post-transplant TCMR in kidney transplant recipients. Moreover, we explored the potential interaction between the HLA-B leader peptide dimorphism and CMV serostatus, hypothesizing that recipient CMV seropositivity may affect leader peptide effects. By examining these factors, we aim to contribute to a deeper understanding of how HLA-B leader peptides may influence kidney transplant outcomes, potentially informing strategies for donor-recipient matching and improving transplant success rates.

## 2 Material and methods

### 2.1 Study population

**Figure 1:**
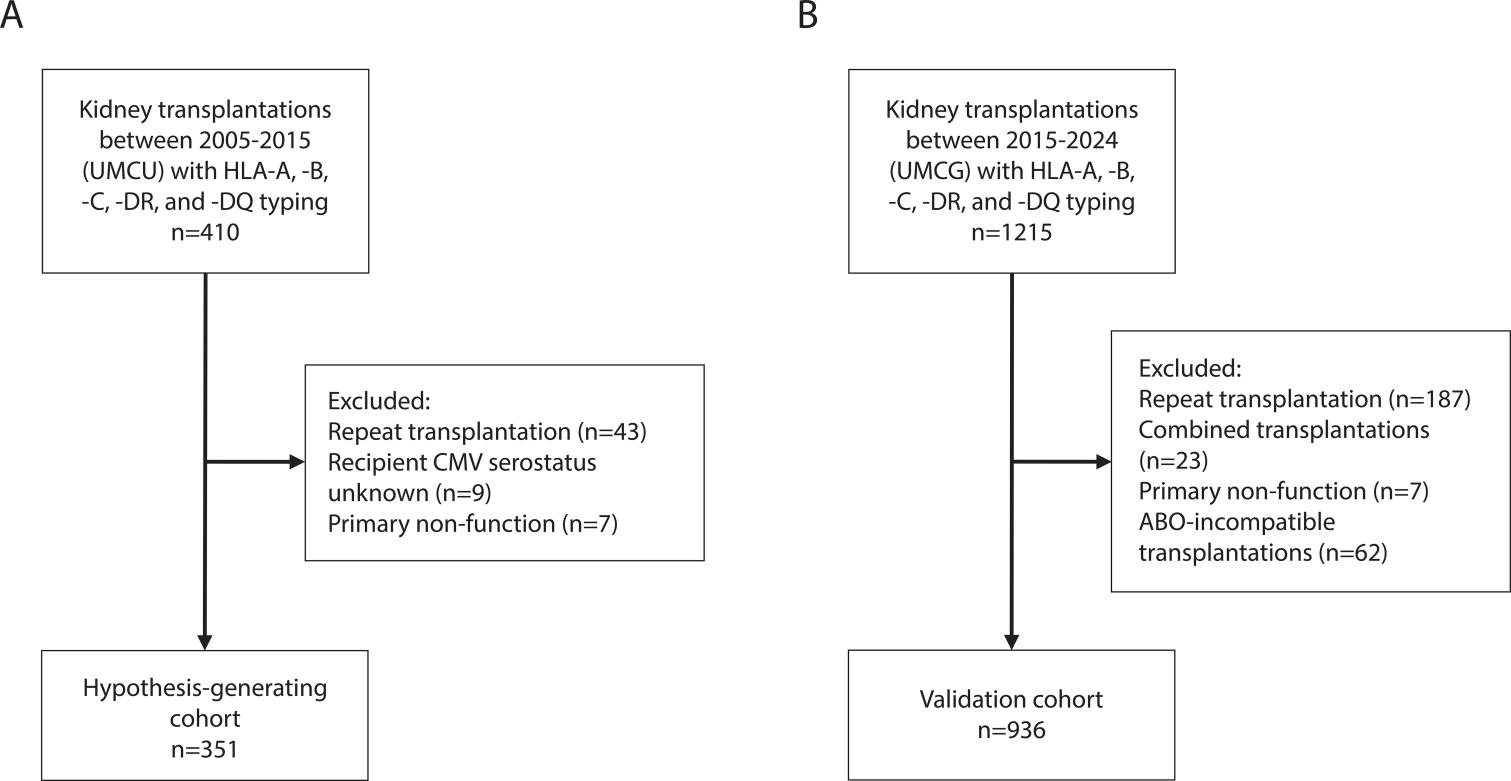
Flow chart with exclusion criteria for the hypothesis generating cohort [**(A)**, n=351] and the validation cohort [**(B)**, n=936].

The hypothesis-generating cohort included patients who received a kidney transplant from a living or a deceased donor in the Utrecht Medical Center Utrecht between 2005 and 2015 (13). Pre-transplant, all patients had a negative T- and B-cell complement-dependent cytotoxicity assay crossmatch. Clinical outcomes were derived from the Dutch Organ Transplantation Registry (NOTR) supplemented with data from the local patient records. All patients had provided informed consent for this registry. The use of clinical data of the subjects involved in this study was approved by the ethics committee for biobanks at the UMC Utrecht (TCBio; reference number: 13-633). Exclusion criteria were: repeat transplantations (n=110), transplantations with unknown CMV serostatus of the recipient (n=20), and transplantations with primary non-function (n=7). A total of 351 kidney transplantations met the inclusion criteria (**Figure 1**). The validation cohort included data from the ongoing TransplantLines Biobank and Cohort study (ClinicalTrials.gov identifier: NCT03272841) (25). The TransplantLines study comprised 1215 kidney transplantations performed in the University Medical Center Groningen, the Netherlands, between January 2015 and December 2024. All participants gave written informed consent on enrollment. The study protocol has been approved by the local Institutional Review Board (METC 2014/077) and adheres to the UMCG Biobank Regulation. After exclusion of repeat transplantations (n=187), combined transplantations (n=23), primary non-function (n=7), and ABO-incompatible kidney transplantations (n=62), the final validation cohort comprised a total of 936 transplantations. CMV serostatus was known for all recipients. This study was conducted in accordance with the Declaration of Helsinki and the Declaration of Istanbul.

### 2.2 HLA typing

For the hypothesis-generating cohort, HLA typing was performed using polymerase chain reaction with sequence-specific oligonucleotides (PCR-SSO) (OneLambda, Canoga Park, CA, USA). For the validation cohort, HLA typing was performed using PCR-SSO (Lifecodes, Norcross, GA, USA) and/or next-generation sequencing (GenDx, Utrecht, the Netherlands). HLA typings were available at intermediate resolution for all included kidney transplant donors and recipients for a minimum of HLA-A, -B, -C, -DR, and -DQ.

### 2.3 HLA-B -21 leader peptide variant assignment

The presence of a methionine or a threonine at position -21 of the HLA-B leader peptides was determined using the HLA-B amino acid sequences deposited in the IPD-IMGT/HLA Database Release 3.58 (2024-10) and the R package HLAtools (DOI: 10.32614/CRAN.package.HLAtools; accessed 31 July 2025). In case only a first-field typing was available, leader peptides were assigned based on allele frequencies. More specifically, the relative frequency of a specific allele within its allele group (first field) was calculated by dividing the frequency of that allele according to the EURO CIWD 3.0.0 data (26) set by the combined frequency of all the alleles within the allele group in that same population. Within each allele group, the amino acid at position -21 was the same for more than 99.5% of the alleles. The remaining 0.5% either had a different amino acid at position -21, or was reported as unsequenced in the IPD-IMGT/HLA Database. Given that over 99.5% of alleles within each group contain the same leader peptide, leader peptides could be assigned for each individual based on first-field typing with an accuracy in at least 0.995 x 0.995 (both alleles) = 99% of the cases. VMAPRTLIL, VMAPRTLLL, and VMAPRTLVL mismatches were determined similarly, based on HLA typing of recipients and donors and HLA-A and -C amino acid sequences deposited in the IPD-IMGT/HLA Database Release 3.58.

### 2.4 Clinical outcomes

The primary end point of this study was biopsy-proven TCMR within the first 90 days after transplantation. In both the hypothesis-generating cohort and the validation cohort, patients with deterioration in kidney function and/or unexplained proteinuria underwent an indication biopsy to validate suspicion of rejection of the transplanted kidney. Borderline rejections were not classified as TCMR in this study.

### 2.5 Statistical analysis

Statistical analyses were performed using R version 4.3.0. The association of the -21 leader peptide dimorphism with TCMR was studied using a logistic regression model. For the validation cohort, the effects of the HLA-B -21 leader peptide genotypes were adjusted for multiple known risk factors associated with allograft rejection and/or graft survival (27). These factors included recipient age (28), recipient sex (29), donor type (deceased vs. living) (30), and donor age (31), the number of HLA-A/B/DR mismatches (27, 32), and the natural logarithm (ln)-transformed PIRCHE-II score as an indicator for the number of donor-derived CD4^+^ T-cell epitopes (33, 34). The number of HLA mismatches was categorized at serological split level as follows: 0 or 1 mismatch, 2 or 3 mismatches, and 4 or more mismatches. PIRCHE-II scores were calculated using the TxPredictor algorithm (version v4.2.87, available via https://www.pirche.com). The proportional hazards assumption was assessed using Schoenfeld residuals. If proportional hazards could be assumed, a log-rank test was performed to compare cumulative TCMR incidences between groups. P-values below 0.05 were considered statistically significant. Holm’s correction was used to correct for multiple testing.

## 3 Results

In the hypothesis-generating cohort, a total of 351 transplant pairs was included (**Table 1**). Early TCMR (within 90 days after transplantation) was diagnosed in 51 recipients (14.5%). The recipients and donors were grouped according to their HLA-B leader peptide genotype (recipients: 8.8% -21MM, 40.7% -21MT, 50.4% -21TT; donors: 12.0% -21MM, 43.0% -21MT, 45.0% -21TT). The validation cohort consisted of 936 transplantations performed in an independent center. Early TCMR was observed in 35 recipients (3.7%). HLA-B leader peptide frequencies were similar to those in the hypothesis-generating cohort (recipients: 12.3% -21MM, 42.5% -21MT, 45.2% -21TT; donor: 10.4% -21MM, 44.6% -21MT, 45.1% -21TT) (p=0.11 for the recipients, p=0.69 for the donors). Induction therapy in the hypothesis-generating cohort consisted primarily of prednisolone, whereas in the validation cohort, mostly prednisolone in combination with basiliximab was given. In both cohorts, maintenance immunosuppression consisted mostly of a triple immunosuppression regimen (tacrolimus, mycophenolate mofetil, and prednisolone).

**Table 1:** Baseline characteristics.

	Hypothesis-generating cohort (n=351)	Validation cohort (n=936)
Recipient characteristics		
Age recipient (median, IQR)	55 (43-64)	60 (49-68)
Sex recipient (male, %)	217 (61.8%)	594 (63.5%)
CMV serostatus recipient (positive, %)	197 (56.1%)	476 (50.9%)
Panel reactive antibodies (PRA) (n, %)^1^		
- <5%	320 (91.2%)	906 (96.8%)
- 5-85%	27 (7.7%)	22 (2.4%)
- >85%	4 (1.1%)	8 (0.9%)
HLA-B leader peptide (n, %)		
- -21MM	31 (8.8%)	115 (12.3%)
- -21MT	143 (40.7%)	398 (42.5%)
- -21TT	177 (50.4%)	423 (45.2%)
Donor characteristics		
Age donor (median, IQR)^2^	55 (47-62)	57 (49-66)
Sex donor (male, %)^3^	157 (44.7%)	492 (52.7%)
CMV serostatus donor (positive, %)^4^	168 (51.1%)	477 (51.0%)
Type donor (living, %)	171 (48.7%)	553 (58.0%)
HLA-B leader peptide (n, %)		
- -21MM	42 (12.0%)	97 (10.4%)
- -21MT	151 (43.0%)	417 (44.6%)
- -21TT	158 (45.0%)	422 (45.1%)
Transplantation characteristics		
Sex mismatch (n, %)^3^		
- Matched	161 (45.9%)	426 (45.7%)
- Female recipient, male donor	65 (18.5%)	204 (21.9%)
- Male recipient, female donor	125 (35.6%)	303 (33.5%)
Transplantation year (median, IQR)	2012 (2010-2014)	2020 (2017 – 2022)
PIRCHE-II score (median, IQR)^5^	59 (33-86)	52 (35 – 69)
TCMR within 90 days post-Tx (n, %)	51 (14.5%)	35 (3.7%)
TCMR during complete follow-up (n, %)^6^	66 (18.8%)	90 (9.6%)

^1^PRA values indicate the maximal PRA values measured before transplant.

^2^23 missing values in the validation cohort.

^3^3 missing values in the validation cohort.

^4^22 missing values in the hypothesis-generating cohort.

^5^2 missing values in the hypothesis-generating cohort, 17 missing values in the validation cohort.

^6^Median follow-up for the hypothesis-generating cohort of 7.8 years, median follow-up for the validation cohort 3.5 years.

### 3.1 Recipient -21M leader peptide significantly associates with early TCMR

**Figure 2:**
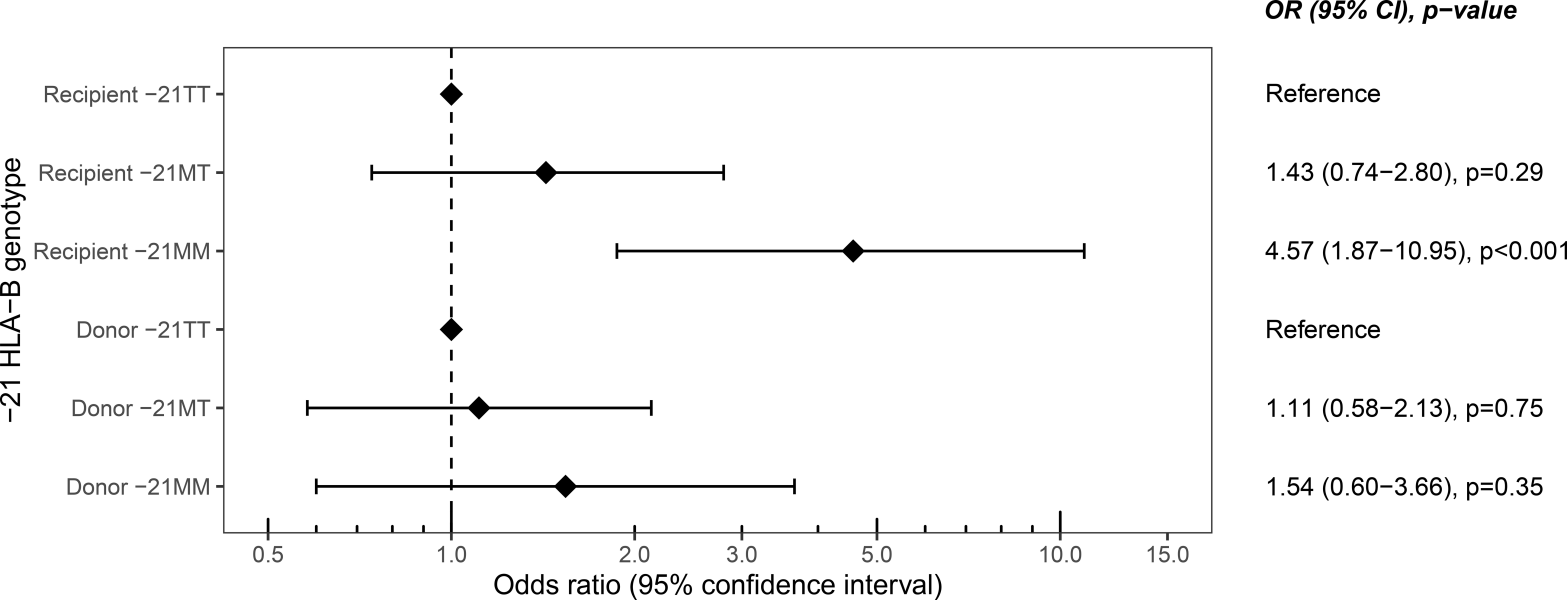
Odds ratios of different recipient and donor -21 HLA-B leader peptide genotypes for T cell-mediated rejection within 90 days after transplantation in the hypothesis-generating cohort. OR, odds ratio; CI, confidence interval.

We first tested whether the HLA-B leader peptide genotype was associated with TCMR in the hypothesis-generating cohort. -21MM recipients had increased odds of developing early TCMR compared to -21TT recipients, with an odds ratio (OR) of 4.57 (95% confidence interval (CI) 1.87-10.95, p<0.001) (**Figure 2**). For TCMR during the full period of follow-up, including TCMR after 90 days post-transplantation, a similar effect was observed, although with a smaller odds ratio (3.66, 95% 1.58-8.41, p=0.002) (**Supplementary Figure 1**).

### 3.2 Effect of -21M leader peptide is independent of HLA-B leader peptide mismatch

To explore whether the significant association between recipient -21M homozygosity (-21MM) and TCMR (**Figure 2**) was attributable to mismatching of the HLA-B leader peptide, we analyzed the combination of the HLA-B leader peptides of the donors and recipients. Compared to matched recipients, recipients who were mismatched at the -21 position of the HLA-B leader peptide did not have significantly increased odds of developing early TCMR (OR 1.72, 95% CI 0.93-3.13, p=0.08). Separating the mismatched recipients for -21M and -21T mismatches yielded similar results. More specifically, comparing the -21MM recipients transplanted with a donor with at least one -21T allele to -21MM recipients matched for the -21 dimorphism gave an OR of 2.31 for early TCMR (95% CI 0.53-12.50, p=0.29). Comparably, no significant effect for early TCMR was observed for -21TT recipients transplanted with a donor with at least one -21M leader peptide compared to -21TT recipients matched for the -21 dimorphism (OR 1.40, 95% CI 0.55-3.50, p=0.47). Combined, these results indicate that HLA-B leader peptide mismatching does not increase the odds of TCMR after a kidney transplantation.

### 3.3 Association between recipient -21MM and early TCMR is most evident in CMV-seropositive recipients

**Figure 3:**
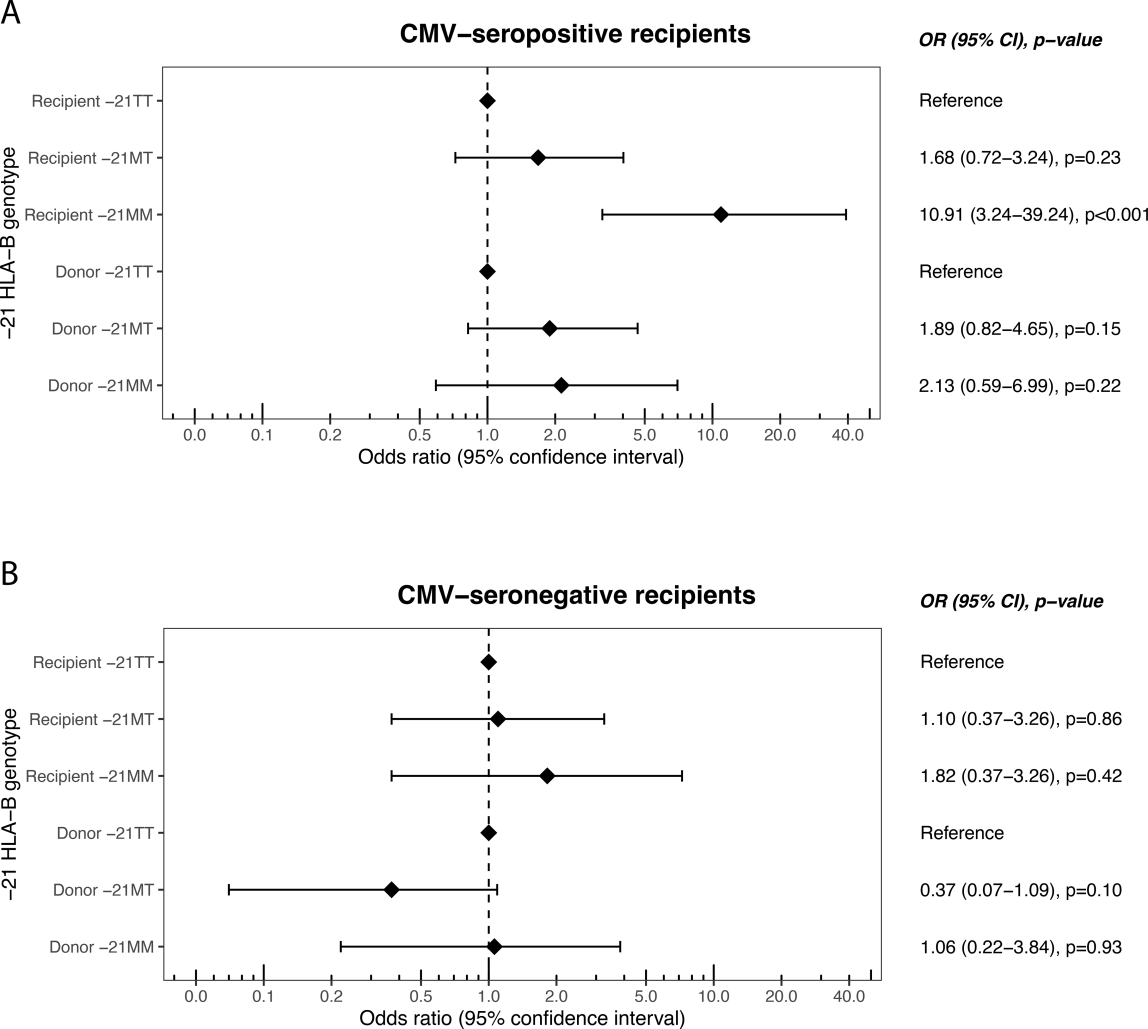
Odds ratios of different recipient and donor -21 HLA-B leader peptide genotypes for T cell-mediated rejection within 90 days after transplantation in the hypothesis-generating cohort, stratified by recipient CMV serostatus. Upper panel: CMV-seropositive recipients. Lower panel: CMV-seronegative recipients. OR, odds ratio; CI, confidence interval.

Next, a potential additional effect of CMV serostatus was examined, in parallel to the relation between HLA-A and -C leader peptides and CMV previously described (13). Hence, the cohort was subdivided into CMV-seronegative and CMV-seropositive recipients. The association between recipient -21MM and early TCMR doubled from an OR of 4.57 (**Figure 2**) in the total cohort to an OR of 10.91 (95% CI 3.24-39.24, p<0.0001) for the CMV-seropositive recipients (**Figure 3**, upper panel). In contrast, we did not find a significant association between the -21 leader peptide genotypes and early TCMR among the CMV-seronegative recipients (**Figure 3**, lower panel).

**Figure 4:**
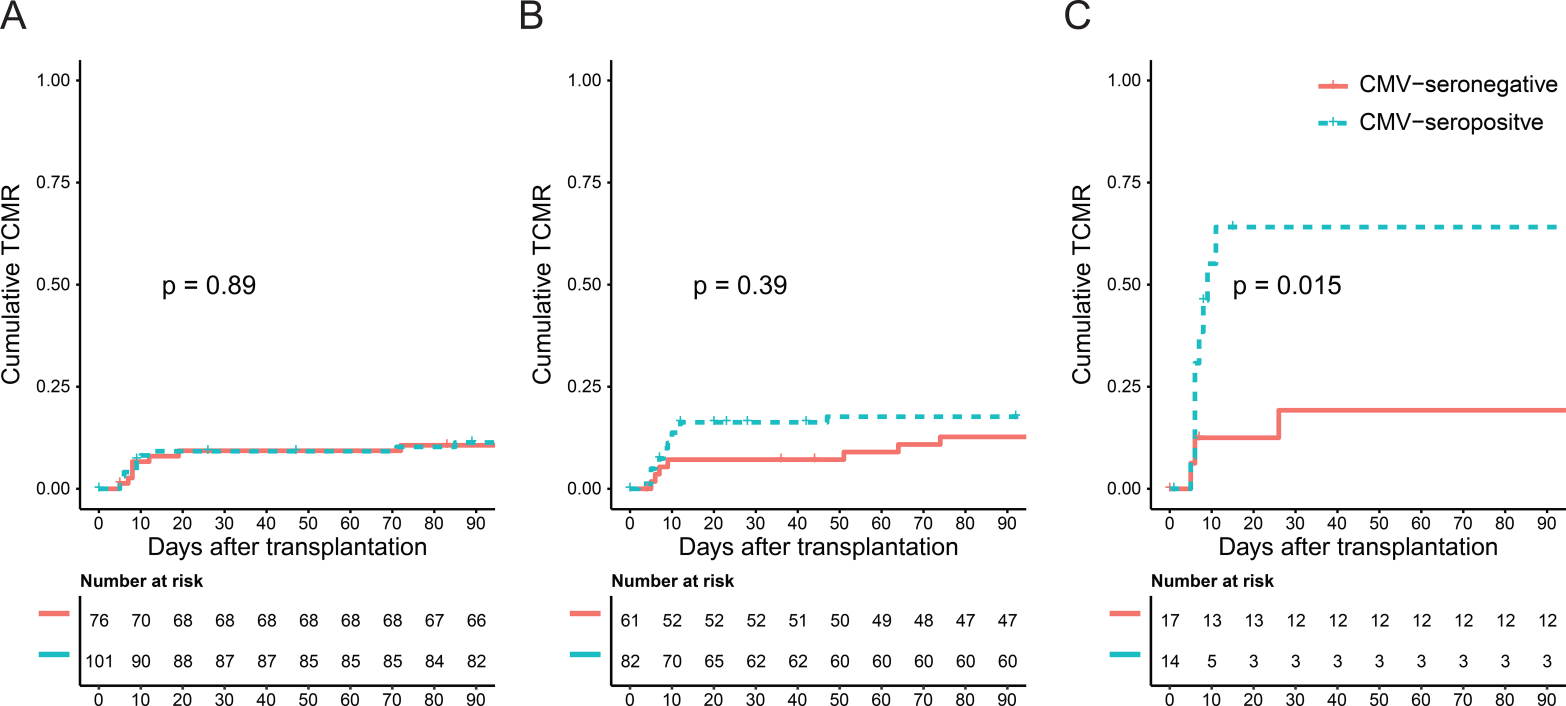
Cumulative T cell-mediated rejection separated for recipient CMV serostatus in the hypothesis-generating cohort, stratified by recipient -21T homozygosity **(A)**, recipient -21MT heterozygosity **(B)**, and recipient -21M homozygosity **(C)**.

When examining the association of recipient CMV serostatus with TCMR over time across the different HLA-B leader peptide genotypes, no association emerged among -21TT recipients (**Figure 4A**). In contrast, a positive CMV serostatus was associated with a prominent and significant rise in cumulative TCMR among -21MM recipients (**Figure 4C**). This rise was exclusively observed in the first few weeks after transplantation. Among -21MT recipients, a similar trend was noted, although the association did not reach statistical significance (**Figure 4B**).

### 3.4 Recipient CMV seropositivity combined with the presence of at least one -21M leader peptide associates with early TCMR

The findings of the hypothesis-generating cohort were validated in an independent validation cohort. Among CMV-seropositive recipients, -21MM recipients seemed to be at increased odds of early TCMR, although this effect was not significant (OR 2.55, 95% CI 0.63-9.23, p=0.16). Yet, the trend aligned with the association found in hypothesis-generating cohort. In addition, -21MT recipients had a significantly increased likelihood of developing early TCMR (OR 2.74, 95% 1.08-7.88, p=0.04). Similar to hypothesis-generating cohort, the associations were absent among CMV-seronegative recipients (**Supplementary Figure 2**).

**Figure 5:**
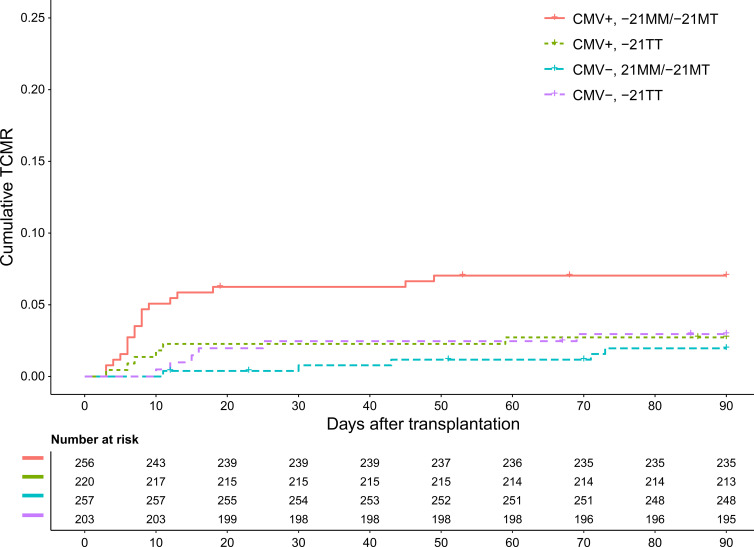
Cumulative T cell-mediated rejection in the validation cohort separated for recipient CMV serostatus and the presence of a -21M leader peptide in the recipient.

Following the significant association between recipient -21MT and CMV seropositivity, as well as the trend found in the hypothesis-generating cohort (**Figures 3A** and **4B**), the -21MM and -21MT recipients were grouped. In both cohorts, there was a significant difference between the two groups regarding recipient age, donor age, and donor CMV serostatus. Other baseline characteristics did not differ between the groups (**Supplementary Tables 1** and **2**). In the hypothesis-generating cohort, CMV-seropositive -21MM/-21MT recipients had increased odds of developing early TCMR compared to recipients who are CMV-seronegative and/or -21TT (OR 2.32, 95% CI 1.25-4.27, p=0.01). Similarly, in the validation cohort, CMV-seropositivity combined with the presence of at least one -21M leader peptide in the recipient was associated with early TCMR with an OR of 2.95 (95% CI 1.49-5.86, p=0.002). In parallel to the hypothesis-generating cohort (**Figure 4**), the rise in early TCMR in the -21MM/-21MT group was observed in the first few weeks after transplantation (**Figure 5**). In the -21TT group, CMV serostatus was not associated with cumulative TCMR in the first 90 days post-transplantation (**Figure 5**, purple and green line). When grouping the hypothesis-generating cohort for the presence or absence of a -21M HLA-B leader peptide, similar effects were observed (**Supplementary Figure 3**).

In both cohorts, the logistic regression model was adjusted for potential confounders. After adjustment for recipient age and sex, donor type (deceased vs. living), donor age, and number of HLA-A/B/DR mismatches, CMV-seropositivity combined with the presence of at least one -21M leader peptide remained significantly associated with early TCMR in the hypothesis-generating cohort (OR 2.78, 95% CI 1.41-5.49, p=0.003), as well as in the validating cohort (OR 3.10, 95% CI 1.54-6.28, p=0.001). After adjusting for ln-transformed PIRCHE-II scores as a measure for histocompatibility instead of classical HLA-A/B/DR mismatches, the combination of CMV-seropositivity and the presence of at least one -21M leader peptide also remained associated with a higher odds of TCMR in both cohorts with a OR of 2.68 (95% CI 1.38-5.19, p=0.003) and 3.29 (95% CI 1.62-6.77, p=0.001), respectively.

### 3.5 HLA-B leader peptide effects are exclusively present in HLA-A/C leader peptide-mismatched recipients

Potential effect modification of HLA-A and HLA-C leader peptides was evaluated in both cohorts, since both HLA-B -21 leader peptide variants and HLA-A/C leader peptides mismatches are associated with early TCMR in CMV-seropositive recipients (13, 35). In the hypothesis-generating cohort, both VMAPRTLIL and VMAPRTLLL leader peptide mismatches were previously observed to be associated with an increased risk for early TCMR in CMV-seropositive individuals (13). Therefore, the cohort was separated into VMAPRTLIL- or VMAPRTLLL-mismatched transplantations and VMAPRTLIL- and VMAPRTLLL-matched transplantations. A significant association between recipient CMV seropositivity combined with the -21M leader peptide and early TCMR was found in the VMAPRTLIL- or VMAPRTLLL-mismatched group exclusively. More specifically, among the recipients mismatched for one of these leader peptides, CMV-seropositivity combined with -21MM/-21MT was associated with early TCMR with an OR of 3.65 (95% CI 1.37-10.34, p=0.01), whereas no significant effect could be observed among recipients matched for VMAPRTLIL and/or VMAPRTLLL (OR 1.14, 95% 0.46-2.58, p=0.76). Cumulative TCMR incidence for the different CMV/HLA-B leader peptide subgroups in the hypothesis-generating cohort for both recipients with and without an HLA-A/C leader peptide mismatch is shown in **Supplementary Figure 4**.

**Figure 6:**
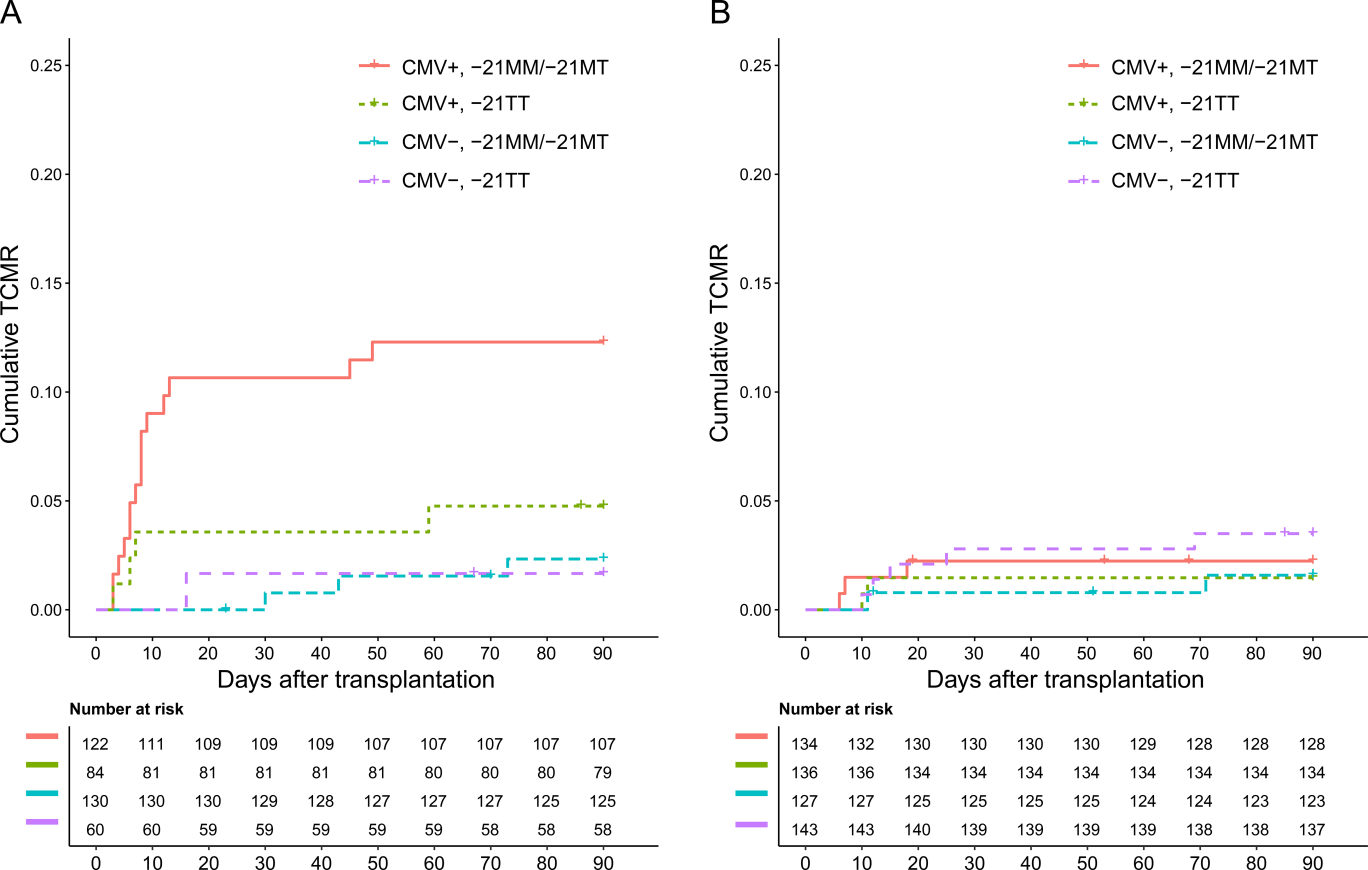
Cumulative T cell-mediated rejection in the validation cohort separated for recipient CMV serostatus and the presence of a -21M leader peptide in the recipient, stratified for HLA-A and HLA-C leader peptide (VMAPRTLIL, VMAPRTLLL, and VMAPRTLVL) mismatches. **(A)** VMAPRTLIL, VMAPRTLLL, and VMAPRTLVL leader peptide-mismatched recipients. **(B)** VMAPRTLIL, VMAPRTLLL, and VMAPRTLVL leader peptide-matched recipients.

In the validation cohort, the VMAPRTLIL, VMAPRTLLL, as well as the VMAPRTLVL leader peptide mismatch were associated with early TCMR (35). Among the recipients having a mismatch for at least one of these HLA-A/C leader peptides (n=396, 42.3%), the positive association between TCMR and the presence of at least one -21M leader peptide in combination with CMV seropositivity was present (OR 4.66, 95% CI 1.97-11.90, p<0.001). In contrast, no association between TCMR and the presence of at least one -21M leader peptide in combination with CMV seropositivity was found in the recipients without an HLA-A/C leader peptide mismatch (OR 1.01, 95% CI 0.22-3.44, p=0.99). Cumulative TCMR incidence for the different CMV/HLA-B leader peptide subgroups in the validation cohort for both recipients with and without an HLA-A/C leader peptide mismatch is shown in **Figure 6**. Notably, the observed association between recipient CMV seropositivity and the presence of a -21M leader peptide in the HLA-A/C leader peptide-mismatched recipients seemed to be exclusively driven by these HLA-A/C leader peptide mismatches and not by any HLA class I leader peptide mismatches, as no effect for CMV and the -21M leader peptide was observed among the HLA-B leader peptide-mismatched recipients (**Supplementary Figure 5**).

## 4 Discussion

The M/T dimorphism at position -21 of HLA-B, located in the leader peptide, affects HSCT outcomes (12, 16, 17, 19–21), but the role of this dimorphism during rejection after kidney transplantation remains elusive. Here, we report that recipients with a -21M variant of the HLA-B leader peptide have an increased likelihood of developing TCMR early after transplantation. We observed that the impact was most prominent in the first two weeks after transplantation, parallelling the early effect of the HLA-B leader peptide on GvHD (12, 21). In addition, for the first time, we report an association between the role of the HLA-B leader peptide during alloreactivity and a positive CMV serostatus. More specifically, we here observed that the odds of developing rejection were twice as high among CMV-seropositive recipients compared to the entire cohort of recipients, indicating an amplification by, or a dependency on, a previous CMV infection in the recipient. The increased odds of early TCMR for CMV-seropositive recipients with a -21M HLA-B leader peptide was confirmed in an independent validation cohort. The higher risk of early TCMR in -21M CMV-seropositive recipients was unrelated to an increased incidence of CMV reactivation in both cohorts (data not shown), although bigger cohorts are needed to examine a potential effect of CMV reactivation in more detail. In both cohorts, a positive CMV serostatus did not increase the odds of early TCMR in -21TT recipients, indicating that a positive CMV serostatus may only affect the odds of TCMR in specific contexts.

HLA-B leader peptide mismatching did not increase the likelihood of TCMR. Strikingly, although independent of HLA-B leader peptide mismatching, the effect of the -21M variant on TCMR seemed to depend on HLA-A/-C leader peptide mismatching. More specifically, the odds ratio increased among the transplantations that were mismatched for specific HLA-A/-C leader peptides that overlap with a sequence in the UL40 protein of specific CMV strains, whereas no significant effect was observed among transplantations matched for these leader peptides. The lack of effect for HLA-B leader peptide mismatching combined with the dependency on HLA-A/-C leader peptide mismatching may indicate that the -21M variant could play a role in the regulation of the immune system, making immune cells more sensitive to leader peptide mismatches. Whether or not the effect of the -21M HLA-B leader peptide variant combined with HLA-A/-C leader peptide mismatching also affects graft survival remains currently unknown, and may be more complicated; treatment strategies most likely change following signs of rejection. These changes in treatment complicate efforts to analyze a direct effect of the HLA-B leader peptide on graft survival.

Conceptually, the underlying immunological mechanism behind the HLA-B leader peptide and transplantation outcomes remains elusive. Notably, the -21T leader peptide binds poorly to HLA-E and leads to lower HLA-E expression in comparison to the -21M variant (36, 37). Accordingly, recipients with a -21M variant, having more NKG2A ligands, have better educated and more functional NK cells as compared to -21TT recipients (38, 39). Upon CMV infection, T cells expressing the activating NKG2C receptor can expand (40–43). The presence of the NKG2C receptor on such CD8^+^ T cells has been suggested as a co-stimulatory molecule, potentiating TCR-mediated cytotoxicity (41, 44). Although these cells may be involved in the rejection episode early after transplantation, better education of NKG2A^+^ NK cells indicates more inhibition upon binding to donor HLA-E. On the other hand, a lower HLA-E expression may lead to missing-self recognition. However, we have no indication that HLA-E is lower expressed in donor cells, as the effect observed for the -21M leader peptide is independent of the leader peptide variant in the donor, also within the subgroup of -21 MM/MT CMV-seropositive recipients with mismatched HLA-A/C leader peptides (data not shown). Our data furthermore suggested a dependency on HLA-A/C leader peptide mismatches. These mismatched leader peptides were shown to strongly bind to and stabilize HLA-E (45). As such, we currently consider it unlikely that donor-specific immune cells leading to early TCMR are activated though missing-self recognition by NK cells.

An alternative explanation for the association between the presence of a -21M HLA-B leader peptide and early TCMR in CMV-seropositive individuals may be provided by Lin et al. (37), who found that the -21M leader peptide induced high HLA-E expression, but conferred lower NKG2A receptor recognition than other leader peptides including VMAPRTLIL and VMAPRTLLL. Consequently, the -21M leader peptide may compete with other leader peptides for binding to HLA-E, thereby potentially decreasing overall activation threshold, potentially including that of immune cells targeting the donor including UL40-specific T cells (46, 47). Yet, the involvement of these cells and whether individuals with the -21M leader peptide have a lower NK-cell activation threshold compared to individuals without this leader peptide warrants further investigation.

Both this study and studies in the field of HSCT have reported that the presence of a -21M leader peptide variant in the recipients can be a risk factor for a poor transplantation outcome (12, 16, 17, 19–21). These findings in the field of HSCT suggest that the HLA-B leader peptide variant is important in the target, but not the effector, of alloreactivity. Although speculative, we theorize that NK-cell education is affected by the leader peptide present in their microenvironment. As both in HSCT and kidney transplantation, this microenvironment is related to the transplant recipient, this hypothesis may resolve the seemingly contradictory findings in these two transplantation settings.

In this study, the two cohorts were analyzed separately to marginalize confounding effects resulting from differences in clinical practices between centers. Indeed, the hypothesis-generating cohort and validation cohort differed substantially based on the incidence of TCMR. Likely, this difference is a consequence of the different time periods in which the transplantations in the two cohorts were performed: the transplantations in the hypothesis-generating cohort were performed significantly earlier than those in the validation cohort. As such, induction therapy was limited in the hypothesis-generating cohort. In addition, patients in this cohort were biopsied in case of delayed graft failure. These biopsies may have been classified incorrectly as rejection. Nonetheless, despite these differences in TCMR incidence, we observed similar effects of the -21M HLA-B leader peptide variant in both cohorts. Notably, combining data from different cohorts into one single outcome analysis requires careful evaluation of cohort-specific differences that may influence the outcomes of interest.

In both cohorts, CMV-seropositive recipients with at least one -21M peptide had significantly more often a CMV-seropositive donor, and both recipients and donors were older in these transplantations. While this difference may be due to the increased likelihood of being CMV-seropositive with age, the effect of CMV seropositivity combined with the -21M leader peptide remained significant after adjustment for age in the validation cohort, indicating a confounding effects of these variables to be unlikely. Furthermore, age of the recipient seems improbable to have confounded our observation that CMV-seropositive -21M^+^ recipients have an increased risk of early TCMR; any potential confounding effect of age would likely bias the results in the opposite direction, given the reduced functionality of alloreactive CD4^+^ and CD8^+^ T cells (48) and a lower incidence of acute rejection (49) among elderly transplant recipients.

The findings in this study could have been affected by unaccounted-for variables including KIR ligands HLA-Bw4/Bw6 and HLA-C1/C2, as well as HLA-E genotypes (12). However, the HLA-B leader peptide dimorphism is unlikely to be a proxy for either of these factors, since Petersdorf et al. observed that the association between HLA-B leader peptide and GvHD could not be explained by KIR ligands or HLA-E genotypes (12). Nonetheless, validation in larger cohorts is required to address the impact of these genotypes in relation to HLA-B leader peptides and transplantation outcomes. Moreover, no genotyping data for exon 1 was present. As such, the amino acid present at position -21 was inferred from intermediate HLA typing, which could have resulted in an incorrect leader peptide variant assignment. However, we here established that within each allele group, over 99.5% of the alleles contain the same leader peptide variant, corresponding to the findings of Sajulga et al. (50) As exceptions are rare, their influence on the leader peptide assignment is therefore minimal.

In conclusion, we show that recipients who are CMV-seropositive and have at least -21M HLA-B leader peptide, which accounts over 25% of the included recipients in the current study, have an increased likelihood of developing TCMR early after transplantation. Although further validation is warranted and questions remain regarding the underlying biological pathway, this study suggests that leader peptide may play a role in immune regulation and the development of TCMR early after a kidney transplantation. If the observation presented in this study can be replicated in other patients with positive CMV serology and -21M HLA-B leader peptide, more rigorous evaluation for possible rejection might be warranted in these patients.

## Data Availability

The original contributions presented in the study are included in the article/**Supplementary Material**. Further inquiries can be directed to the corresponding author.
