# Supplementary material for: Interaction between HLA-B leader peptide variants and cytomegalovirus serostatus is associated with early T cell-mediated rejection in kidney transplantation
Source: Front Immunol. 2026 Feb 9;17:1713932. doi: 10.3389/fimmu.2026.1713932 (PMC12926155; doi:10.3389/fimmu.2026.1713932)
Supplement: Supplementary file 1 [file DataSheet1.pdf]

Supplementary material

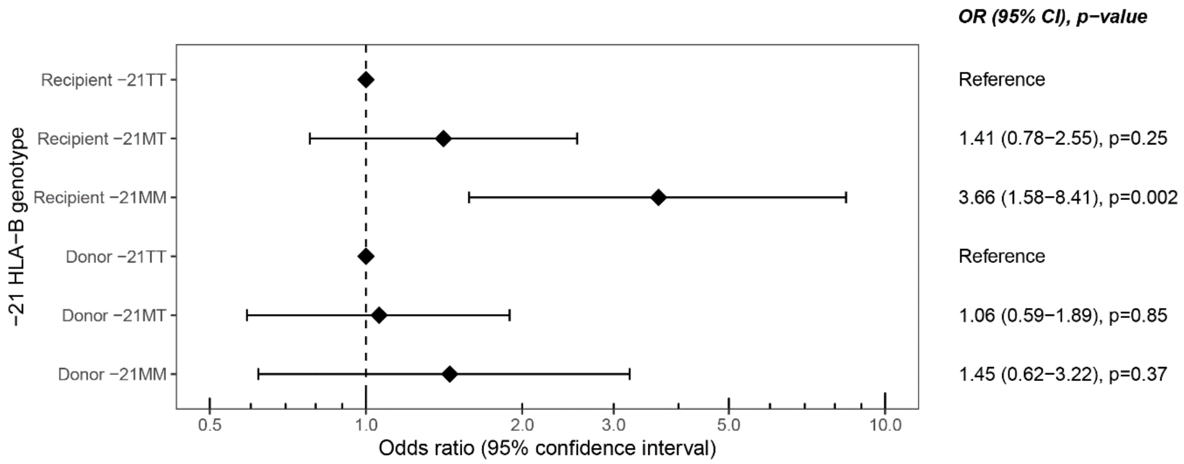

Supplementary Figure 1. *Odds ratios of different recipient and donor -21 HLA-B leader peptide genotypes for T cell-mediated rejection after transplantation in the hypothesis-generating cohort. OR: odds ratio; CI: confidence interval.*

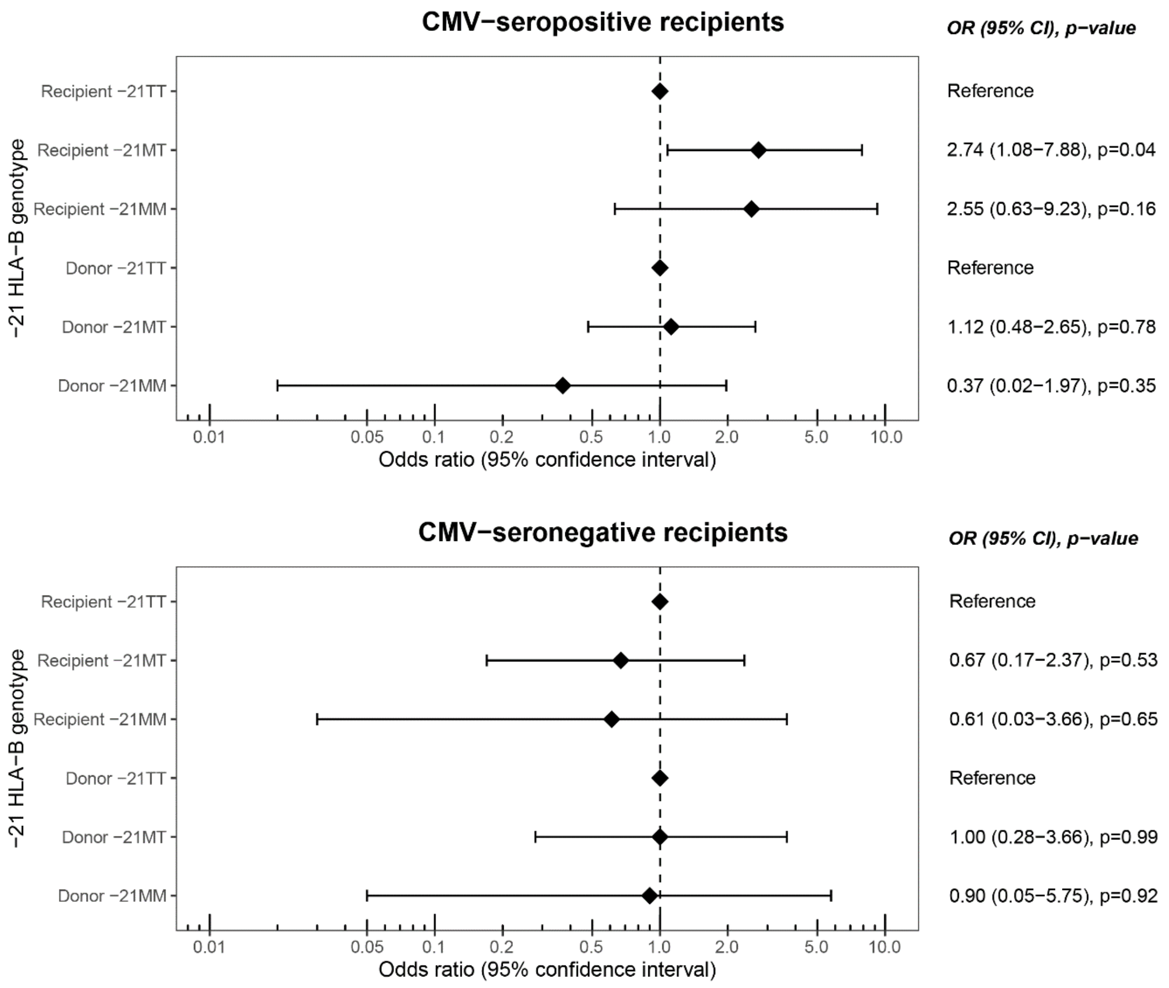

Supplementary Figure 2. *Odds ratios of different recipient and donor -21 HLA-B leader peptide genotypes for T cell-mediated rejection within 90 days after transplantation in the validation cohort, stratified by recipient CMV serostatus. Upper panel: CMV-seropositive recipients. Lower panel: CMV-seronegative recipients. OR: odds ratio; CI: confidence interval.*

Supplementary Table 1. *Baseline characteristics for the hypothesis-generating cohort, stratified by CMV-seropositive and HLA-B -21M leader peptide-positive recipients vs. the rest. P-values in bold indicate statistical significance.*

	**CMV^{+} -21MM/-21MT recipients (n=96)**	**CMV^{-} and/or -21TT recipients (n=255)**	**P-value**
Recipient characteristics			
Age recipient (median, IQR)	59 (50-66)	53 (42-62)	**<0.001**
Sex recipient (male, %)	56 (58.3%)	161 (63.1%)	0.48
CMV serostatus recipient (positive, %)	96 (100%)	101 (39.6%)	N.A.
Panel reactive antibodies (PRA) (n, %)[1]			0.50
- <5%	85 (88.5%)	235 (92.2%)	
- 5-85%	10 (10.4%)	17 (6.7%)	
- >85%	1 (1.0%)	3 (1.2%)	
Donor characteristics			
Age donor (median, IQR)	58 (50-65)	54 (47-61)	**0.01**
Sex donor (male, %)	49 (51.0%)	108 (42.4%)	0.18
CMV serostatus donor (positive, %)[2]	54 (61.4%)	114 (47.3%)	**0.03**
Type donor (living, %)	50 (52.1%)	121 (47.5%)	0.51
Transplantation characteristics			
Sex mismatch (n, %)			0.03
- Matched	37 (38.5%)	124 (48.6%)	
- Female recipient, male donor	26 (27.1%)	39 (15.3%)	
- Male recipient, female donor	33 (34.4%)	92 (36.1%)	
Transplantation year (median, IQR)	2012 (2011-2013)	2012 (2010-2014)	0.73
PIRCHE-II score (median, IQR)[3]	55 (30-74)	60 (34-88)	0.23

[1] *PRA values indicate the maximal PRA values measured before transplant.*

[2] *8 missing values in CMV^{+} -21MM/-21MT recipients, 14 missing values in CMV^{-} and/or -21TT recipients.*

[3] *1 missing value in CMV^{+} -21MM/-21MT recipients, 1 missing value in CMV^{-} and/or -21TT recipients.*

Supplementary Table 2. *Baseline characteristics for the validation cohort, stratified by CMV-seropositive and HLA-B -21M leader peptide-positive recipients vs. the rest. P-values in bold indicate statistical significance.*

	**CMV^+ -21MM/-21MT recipients (n=256)**	**CMV^- and/or -21TT recipients (n=680)**	**P-value**
Recipient characteristics			
Age recipient (median, IQR)	64 (54-70)	58 (47-67)	**<0.001**
Sex recipient (male, %)	166 (64.8%)	428 (62.9%)	0.59
CMV serostatus recipient (positive, %)	260 (100%)	220 (32.4%)	N.A.
Panel reactive antibodies (PRA) (n, %)[1]			0.72
- <5%	248 (96.9%)	658 (96.8%)	
- 5-85%	5 (2.0%)	17 (2.5%)	
- >85%	3 (1.2%)	5 (0.7%)	
Donor characteristics			
Age donor (median, IQR)[2]	60 (52-67)	57 (48-65)	**0.003**
Sex donor (male, %)[3]	141 (55.1%)	351 (51.8%)	0.38
CMV serostatus donor (positive, %)[4]	161 (62.9%)	316 (46.5%)	**<0.001**
Type donor (living, %)	133 (52.0%)	386 (56.8%)	0.19
Transplantation characteristics			
Sex mismatch (n, %)[3]			0.81
- Matched	113 (44.1%)	313 (46.2%)	
- Female recipient, male donor	59 (23.0%)	145 (21.4%)	
- Male recipient, female donor	84 (32.8%)	219 (32.3%)	
Transplantation year (median, IQR)	2020 (2017-2022)	2019 (2017-2022)	0.13
PIRCHE-II score (median, IQR)	50 (33-66)	51 (35-67)	0.71

[1] *PRA values indicate the maximal PRA values measured before transplant.*

[2] *5 missing values in CMV^+ -21MM/-21MT recipients, 18 missing values in CMV^- and/or -21TT recipients.*

[3] *3 missing values in CMV^- and/or -21TT recipients.*

[4] *2 missing values in CMV^+ -21MM/-21MT recipients, 15 missing values in CMV^- and/or -21TT recipients.*

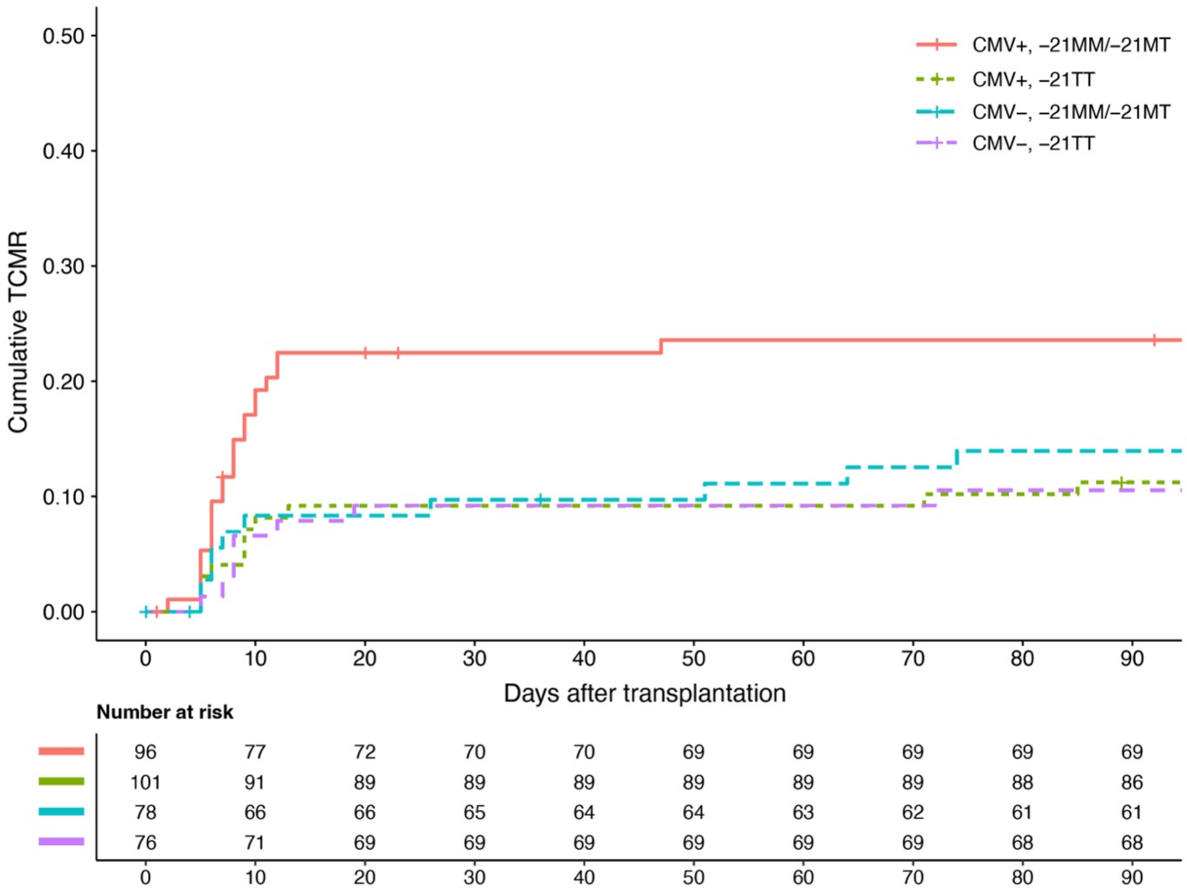

Supplementary Figure 3. *Cumulative T cell-mediated rejection in the hypothesis-generating cohort separated for recipient CMV serostatus and the presence of a -21M leader peptide in the recipient.*

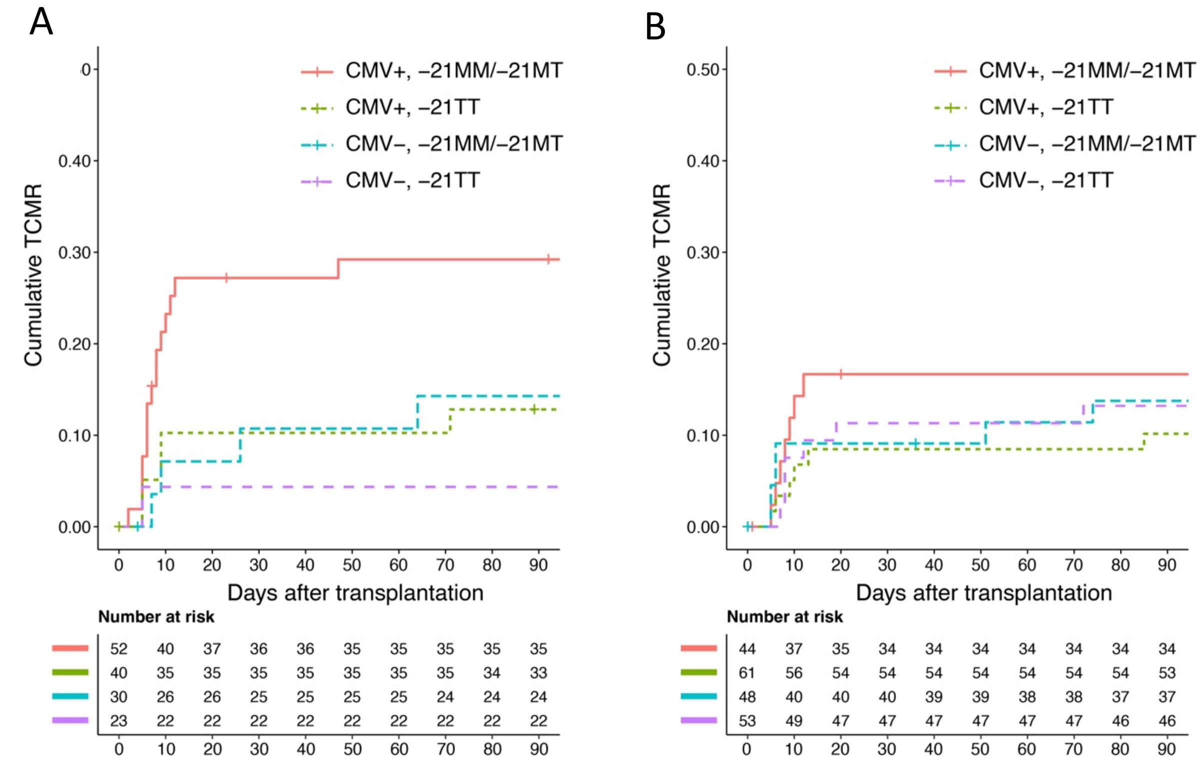

Supplementary Figure 4. *Cumulative T cell-mediated rejection in the hypothesis-generating cohort separated for recipient CMV serostatus and the presence of a -21M leader peptide in the recipient, stratified for HLA-A and HLA-C leader peptide (VMAPRTLIL, VMAPRTLLL, and VMAPRTLVL) mismatches.* ***(A)*** *VMAPRTLIL, VMAPRTLLL, and VMAPRTLVL leader peptide-mismatched recipients.* ***(B)*** *VMAPRTLIL, VMAPRTLLL, and VMAPRTLVL leader peptide-matched recipients.*

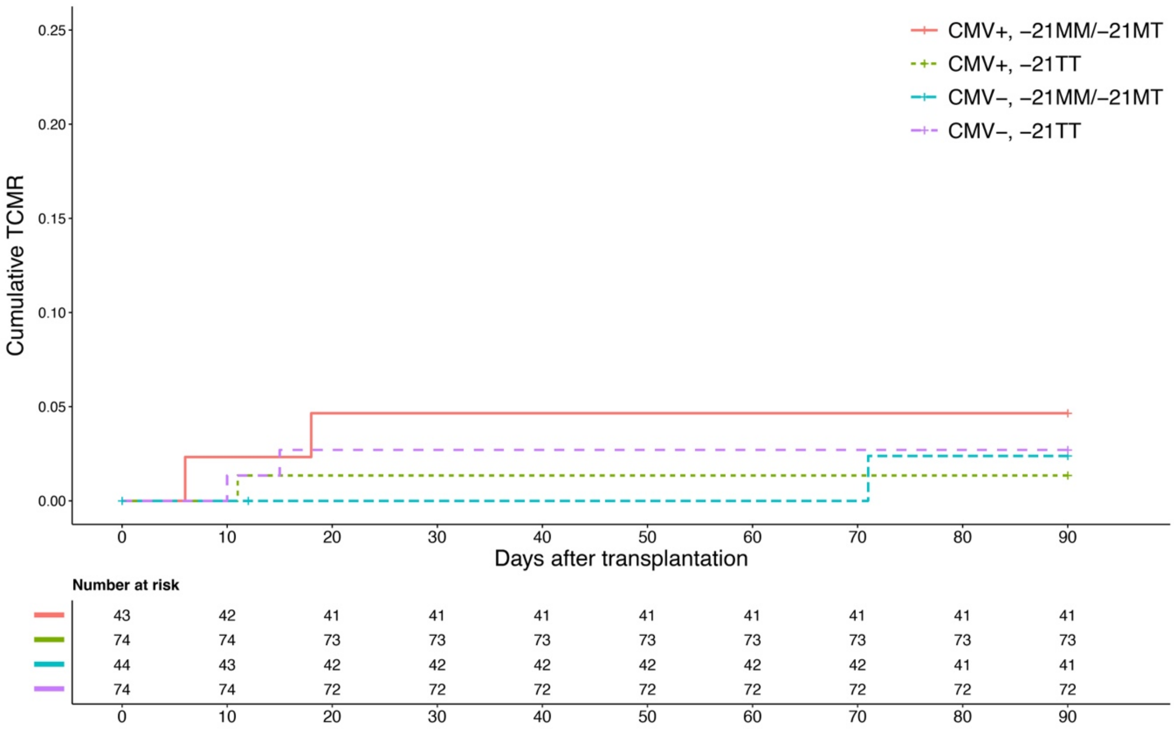

Supplementary Figure 5. *Cumulative T cell-mediated rejection in the subgroup of HLA-A/C leader peptide-matched, HLA-B leader peptide-mismatched recipients in the validation cohort, separated for recipient CMV serostatus and the presence of a -21M leader peptide in the recipient.*
